# Creativity in lockdown: Understanding how music and the arts supported mental health during the COVID-19 pandemic by age group

**DOI:** 10.3389/fpsyg.2022.993259

**Published:** 2022-10-06

**Authors:** Anthony Chmiel, Frederic Kiernan, Sandra Garrido, Sarah Lensen, Martha Hickey, Jane W. Davidson

**Affiliations:** ^1^Melbourne Conservatorium of Music, University of Melbourne, Melbourne, VIC, Australia; ^2^The MARCS Institute for Brain, Behaviour and Development, Western Sydney University, Sydney, NSW, Australia; ^3^Creativity and Wellbeing Hallmark Research Initiative (CAWRI), University of Melbourne, Melbourne, VIC, Australia; ^4^Department of Obstetrics and Gynaecology, University of Melbourne, Melbourne, VIC, Australia; ^5^Royal Women’s Hospital, Melbourne, VIC, Australia

**Keywords:** COVID-19, Australian lockdown, mental health, artistic creative activities, young people, age, anxiety and depression, exercise

## Abstract

Recent research has indicated that many people around the world turned to artistic creative activities (ACAs) to support their mental health during COVID-19 lockdowns. However, studies have also shown that the negative psychosocial impacts of the pandemic have disproportionately affected young people, suggesting that the use of ACAs to support mental health in lockdown may have varied across age groups. This study investigated how Australians in four different age groups (18–24, 25–34, 35–54, and 55+) engaged in ACAs to support their mental health during the 2020 pandemic lockdowns. Participants (*N* = 653) recruited from the general public completed an online survey between May and October 2020, in which they reported which ACAs they had undertaken during the pandemic using an established list. Participants subsequently ranked undertaken ACAs in terms of effectiveness at making them “feel better,” and those who had engaged in musical ACAs also completed the Musical Engagement Questionnaire (MusEQ). Additionally, ratings of anxiety (GAD7) and depression (PHQ9) symptoms were obtained and examined for potential variations across four diverse variables: age, exercise, gender and state/territory of residence. ACA rankings showed that younger participants overwhelmingly rated musical activities as most effective, while, with the exception of singing, those aged 55+ rated non-musical activities as being most effective. These differences were further supported by ratings for all six MusEQ subscales, with responses strongly indicating that the youngest participants (aged 18–24) used music in significantly different ways during the pandemic than participants in all other age groups. Specifically, these youngest participants were more likely to integrate music into daily life, use music for emotion regulation, respond to music in embodied ways, and use music to perform a social identity. In line with prior research, further analyses indicated that symptoms of anxiety and depression were lessened for older participants, as well for those who reported exercising more during the lockdowns. These findings provide insight into how ACAs can support mental health during a pandemic crisis for specific age groups, which may inform future policy directions, and suggestions for how this can be done are provided.

## Introduction

An extensive body of research literature has emerged examining the variety of ways engagement in music and other creative arts has supported mental health during the COVID-19 pandemic, in addition to non-creative activities such as increasing the amount of time spent in nature (Ribeiro et al., 2021) or increasing exercise (Colley et al., 2020; Coyle et al., 2021; Faulkner et al., 2021). A recent special issue of *Frontiers in Psychology* has captured 44 studies on the topic of “Social convergence in times of spatial distancing: The role of music during the COVID-19 pandemic” (Hansen et al., 2022), and a forthcoming review of this literature has broadly shown that music has supported both eudaimonic and hedonic aspects of wellbeing during the pandemic (Hansen, forthcoming). Population-level studies have also been undertaken, with one Spanish study finding that music has helped people to relax, escape, lift their mood, and keep themselves company (Cabedo-Mas et al., 2021), while an Australian study (Kiernan et al., 2021) examined how *artistic creative activities* (henceforth ACAs, defined as creative activities specifically involving the arts; see Fancourt et al., 2019) helped participants regulate emotion during pandemic lockdown, primarily through avoidance-based emotion-regulation strategies.

A similar, recent study which recruited a majority (90%) of participants from the United States also found music listening to be the most helpful ACA for coping with the pandemic, and that ACAs most commonly regulated emotions by providing a means of avoidance/escape (Drake et al., 2022). Drake et al. (2022) further reported that the personality trait of “openness to experience” predicted the use of approach-and self-development-based emotion regulation strategies. Other studies on the role of music and the arts in responding to the pandemic have examined how social-distancing measures and stay-at-home orders encouraged engagement in both active and receptive creative activities such as baking (Easterbrook-Smith, 2021), watching films (Mikos, 2020), visiting online art galleries (Shehadi, 2020), and singing in online choirs or from balconies (Taylor, 2020). These have served different social purposes such as raising awareness about the threat of the virus (Blanc et al., 2020) and memorializing (de-Graft Aikins, 2020). This research highlights the variety of ways that engagement in music and the arts has supported mental health and wellbeing during the pandemic for different individuals and social groups globally.

Australia reported substantially lower rates of COVID-19 infection and death in 2020 than many other countries, particularly when compared to much of Europe, the United Kingdom, and the United States (Berger and Reupert, 2020; Pew Research Center, 2020; Hong et al., 2021). This comparative success in controlling the spread of the virus hinged largely on two extended periods of lockdown then reported to be among the most restrictive in the world (BBC News, 2020). The first lockdown was enforced nationwide for a duration of 8 weeks from late March 2020, while the second was enforced in the state of Victoria from early July until late October 2020, with varying degrees of restriction across the almost four-month period, in response to a second wave of infection (Knowlton, 2022). Yet while these lockdowns protected many from infection, the negative mental health impacts have been substantial (Fisher et al., 2020, 2021; Australian Institute of Health and Welfare, 2022). In particular, recent research in Australia has highlighted the significant impact of the pandemic on the mental health and wellbeing of young adults^1^ (Biddle et al., 2022), corroborating the findings of previous international research (Power et al., 2020; Rousseau and Miconi, 2020; Chahal et al., 2021; Penner et al., 2021). Researchers have argued that some of these impacts relate to fears of unemployment and limited job security (Power et al., 2020; Mak et al., 2021), the availability of family support and internal dynamics of family functioning (Penner et al., 2021; Zolopa et al., 2022), and increased caregiving responsibilities in the family (Rousseau and Miconi, 2020; Ruff and Linville, 2021).

This research suggests that further investigation is needed into the ways young adults in Australia have used ACAs to support their mental health during the pandemic, and how this may or may not differ from the use of ACAs by older people, as this may help inform the way music and the arts can be strategically engaged in age-appropriate ways to support mental health and wellbeing in future crises. Music-making during the pandemic has been found to help young people restore lost musical identities and preserve social connections (Levstek and Banerjee, 2021), building on extensive previous research which emphasizes the importance of music in identity formation for young people (Tarrant et al., 2002; O’Neill, 2017). Music listening has also been found to be as effective a stress coping strategy as exercise and sleep for first-year university students (Vidas et al., 2021). However, music therapists have highlighted that while young people may use music to facilitate private reflection as a means for working through challenging life experiences, this may not necessarily lead to health promotion. This may especially be the case in contexts where, for example, an individual is experiencing mental illness, has adopted an “illness identity”, and lacks opportunities for expanded notions of self that include aspects of health (Hense and McFerran, 2017). In such contexts, therapeutically facilitated music encounters may be advisable (McFerran and Saarikallio, 2014). This research also suggests that using music for health promotion (both within and outside of therapeutic contexts) is not as straightforward as simply increasing music engagement, and that further research is needed into the ways music can support young people during times of crisis.

## Aims and hypotheses

Based on the paucity of work in this area, the principal aim of this research was to better understand how young adults (aged 18–24) in Australia used music and the arts to support their mental health during the pandemic lockdowns of 2020. The secondary aim was to identify potential similarities and differences in the use of music and the arts for mental health purposes across different age-groups, in order to understand how engagement in ACAs may have varied in Australia by age-group and to further illuminate how music and the arts can be used to meet specific mental health needs during a pandemic crisis. Targeted age groups were young adults (aged 18–24), younger established adults (aged 25–34), established adults (aged 45–54), and older adults (aged 55+),

As a third aim, we also wanted to examine the extent to which a diverse groups of variables were able to predict ratings of mental health, as these data are lacking in Australian research. The demographic variables age and gender were selected due to the prevalence of studies examining these variables alongside mental health in other countries. We additionally included location within Australia (i.e., state/territory) as a variable of interest considering that despite the significant differences in lockdown restrictions around Australia (BBC News, 2020) there has been a lack of prior studies directly investigating how this might impact mental health. Furthermore, as part of this study aimed to examine how creative engagement (i.e., through ACAs) might be able to provide protective benefits for mental health during the COVID-19 pandemic, we also wanted to examine a non-creative equivalent. Due to a growing body of international work that suggests increased exercise may be a robust method for maintaining mental health (albeit again with a significantly smaller focus examining Australian responses) we decided to also include this variable for comparison. With these points in mind, we investigated the following research questions:RQ1: Which ACAs do people in Australia aged 18–24, 25–34, 35–54, and over 55 report as being the most effective at making them “feel better” during the COVID-19 pandemic?RQ2: What similarities and differences can be observed in the ways people in Australia aged 18–24, 25–34, 35–54, and over 55 have engaged in musical activities specifically during the COVID-19 pandemic?RQ3: Which of three demographic variables (age, gender, state/territory) and a fourth non-creative protective variable (exercise) best predict variance in mental health?Based on the findings of the literature discussed in the introduction, especially the findings of Kiernan et al. (2021) and Drake et al. (2022), our preliminary hypothesis (H1) was that music listening would rank as the most effective ACA for making people “feel better” during the pandemic in each of the four age groups. Based on the findings of Tarrant et al. (2002), O’Neill (2017) and Levstek and Banerjee (2021), our secondary hypothesis (H2) was that participants aged 18–24 would be more likely to use music for the purposes of identity expression than older participants during the pandemic. Additionally, based on the findings of Biddle et al. (2022), we hypothesized (H3) that young adults, women, and residents of Victoria would report poorer mental health (i.e., increased symptoms of anxiety and depression) than other participants.

## Materials and methods

Data were gathered for this study using an online, cross-sectional survey of people aged 18 and over residing in Australia, that was made available between 29th May 2020 and 16th October 2020 inclusive. More details are provided in the *Procedure* section.

### Participants

A total of 952 participants from the general population responded to the online survey. Of these 952 responses, 267 (28%) were incomplete and so were removed from the dataset. Furthermore, 32 responses (3.4% of the overall sample) were made from participants living outside of Australia. These responses were also removed from the dataset, in line with the research focus on the mental health of Australians and to keep the sample as homogenous as possible. This led to a final sample of *N* = 653. Five-hundred and fifty-one participants (84.4%) self-identified as female, 87 (13.3%) self-identified as male, 6 (0.9%) selected “other,” and 9 (1.4%) selected “prefer not to say.” Participants also indicated which Age group they belonged to. One-hundred and twenty-seven participants (19.4%) were aged 18–24, 127 (19.4%) were aged 25–34, 144 (22%) were aged 35–44, 150 (23%) were aged 45–54, 75 (11.5%) were aged 55–64, and 30 (4.6%) were aged 65 and older. These specific age groups were chosen due to their frequency in existing studies on mental health both during the COVID-19 pandemic (e.g., see studies cited in our Introductory section) as well as studies from years earlier (e.g., Berkman, 1971; Bijl et al., 1998; Rohrer et al., 2005).

The sample covered all eight Australian states and territories. The majority of participants were from Victoria (446, or 68.3%) or New South Wales (83, or 12.7%). Six participants were from the Australian Capital Territory (0.9%), three were from the Northern Territory (0.5%), 33 were from Queensland (5%), 28 were from South Australia (4.3%), 14 were from Tasmania (2.1%), and 37 were from Western Australia (5.7%). To aid with analysis, all states and territories apart from New South Wales (henceforth NSW) and Victoria (henceforth VIC) were collapsed into a third group titled “Other states and territories” (henceforth OST). Overall, this variable is henceforth referred to as “state” for brevity. Within the Supplementary Material we provide a breakdown of ethnicity, income level, and infection rate for the two states NSW and VIC, as well as for Australia overall.

### Procedure

The survey was created with *Qualtrics* and was distributed using an online link *via* mailing lists, websites, and social media platforms. Participants remotely completed the survey on a personal device (computer, tablet, or smartphone). Before starting the survey, all participants read through a plain language statement and a consent form; this study received approval from the Human Research Ethics Committee of the University of Melbourne (Ethics ID 2056873.1). All participants provided informed consent before commencing the survey, and the survey could be stopped at any point after commencement. Participation was voluntary, and participants could withdraw at any stage after commencement. After completing the survey, participants were invited to provide contact details to enter the draw for a $200 AUD gift card. The survey took approximately 20 min to complete.

The survey questions were divided into three blocks—titled “demographics,” “artistic creative activities,” and “wellbeing”—which were presented to participants in randomized order. The ACAs block asked participants to indicate which ACAs, if any, they had been undertaking since the pandemic began. If the participant selected at least one musical activity (singing; playing a musical instrument; composing music or songs; rehearsing or performing in a play, drama, opera, or musical theatre; dancing; listening to music), they were asked to complete the MusEQ scale (Vanstone et al., 2016). This measure was completed only once regardless of how many musical ACAs the participant selected. In the demographics block, participants were asked to state their age, gender, and country of residence. In this section participants were also asked “Are you doing more, less, or the same amount of exercise during the COVID-19 pandemic?” and were directed to select one of the following responses: “I am exercising less,” “I am exercising the same amount,” or “I am exercising more.”

Regarding age, prior to analysis the decision was made to collapse some of the older age groups to increase statistical power as well as to reduce the number of required hypothesis tests. This was particularly necessary for the 65+ age group, for which *n* = 30. This grouping was only done to older age groups, whereas the two youngest age groups (being the groups most central to the focus of this work) were left untouched. Therefore, for all subsequent analyses the following four age groups were used: 18–24; 25–34; 35–54; 55+. Similarly, those in the “other” and “prefer not to say” gender groups were subsequently grouped into a collapsed group due to the small number of responses (“Other/Prefer not to say,” *n* = 15).

### Measures

Data concerning ACAs undertaken during the pandemic were collected using a list of 26 ACAs, based on that used by Fancourt et al. (2019) and Kiernan et al. (2021); for the full list of ACAs, with theoretical justifications and inclusion/exclusion criteria, see Kiernan et al. (2021).^2^ To collect data about the types of musical activities participants had been engaging in during the pandemic, if any, the 35-item Music Engagement Questionnaire was used (MusEQ; see Vanstone et al., 2016). The MusEQ scale contains six separate subscales, as follows: (1) the “Daily” subscale is made up of a weighted aggregate that addresses the role of music in daily life routines; (2) the “Emotion” subscale concerns music’s role in emotion and mood regulation; (3) the “Perform” subscale addresses the social “performance” of a musical identity; (4) the “Consume” subscale concerns consumer choices in relation to music; (5) the “Respond” subscale concerns embodied responses to heard music (e.g., foot tapping or humming); and, (6) the “Prefer” subscale addresses preferences for certain styles of music (Vanstone et al., 2016). The original authors provide a calculation tool within the Supplementary Material of their publication (Vanstone et al., 2016), with calculations for each subscale made from a weighted aggregate of between three and eight items. Five of the six MusEQ subscales were reported by Vanstone and colleagues to have ‘high’ levels of internal consistency (ranging 0.78–0.82) whereas the “Prefer” subscale scored ‘good’ levels of internal consistency (0.65).

Two standardized measures of mental health were gathered: (1) the 7-item General Anxiety Disorder Scale (GAD7) scale (Spitzer et al., 2006) for assessing symptoms of anxiety and (2) the 9-item Patient Health Questionnaire Depression (PHQ9) scale (Kroenke et al., 2001) for measuring depressive symptoms. Both mental health scales collect responses for each item on a 4-point scale (ranging “Not at all”; “Several days”; “More than half the days”; “Nearly every day,” from least to most symptoms respectively). Spitzer et al. (2006) reported GAD7 as containing “excellent” internal consistency (0.92), and similarly Kroenke et al. (2001) reported “excellent” consistency for PHQ9 (0.89). These were intended to give a brief snapshot of the mental health of participants during the COVID-19 pandemic, although the authors acknowledge that these scales do not cover the entire breadth of this area. The GAD7 and PHQ9 scales each produce an aggregate ranging from 0 to 21 and 0 to 27, respectively. As these scales were designed as a general assessment of severity for anxiety and depression, in their original format the minimum rating of “0” reflects minimal anxiety or depression, and the highest levels of these traits are intended to be scored with the maximum possible ratings (21 and 27). However, in the present paper we inverted both of these scales for ease of analyses and general observations with other data. Therefore, in the reported results for this paper, GAD7 ranged from 0 (most anxiety) to 21 (least anxiety) and PHQ9 ranged from 0 (most depressive symptoms) to 27 (least depressive symptoms). In other words, both variables could be considered as ranging from feeling worst (minimum rating) to feeling best (maximum rating).

## Results

### Examination of artistic creative activities by age group

The ranking of the 26 ACAs for each of the four age groups is reported in Table 1, with Rank 1 equating to the most effective at making participants “feel better.” This list was calculated from the Mean of the Rank score, being the most effective approach to counteract the different sample sizes between the four age groups: 18–24 (*n* = 127), 25–34 (*n* = 127), 35–54 (*n* = 294), 55+ (*n* = 105). The Rank score refers to overall ratings that each ACA received from each participant, from most to least effectiveness at facilitating mental health (i.e., from 1 to 26). The Mean of this Rank score was calculated for each ACA (from the larger list of overall rankings, which are extensive and so are not reported), and then compared with one another. The ACA with the lowest score in the “Mean of Rank” column was taken as the most effective ACA. Based on this comparison, the three youngest age groups (18–24; 25–34; 35–54) ranked “Listening to music” as the most effective ACA. “Singing” was also ranked among the top four ACAs across all four age groups. Similarly, “Dancing” consistently ranked in the top five ACAs for the three youngest age groups (ranked third highest for both the 18–24 and 25–34 age groups, but falling to fifth place for the 35–55 group). In sum, music-based ACAs (if dancing is assumed to involve music) accounted for three of the top five rankings for both the 18–24 and the 35–54 groups, and accounted for four of the top five ACAs for the 25–34 group.

**Table 1:** Ranking of ACAs by reported effectiveness at helping participants to “feel better” during the 2020 COVID-19 lockdowns, split by the four age groups (18–24; 25–34; 35–54; 55+).

Overall rank	Activity	Min. rank	Max. rank	Mean of rank	SD of rank	Rank variance	ACA count
**Participants aged 18–24 (*n* = 127)**							
1	Listening to music	1	13	3.44	2.45	6.01	109
2	Singing	1	9	3.68	2.6	6.75	53
3	Dancing	1	13	3.88	3.07	9.41	43
4	Cookery or baking	1	13	4.36	2.64	6.97	76
5	Creative writing (e.g., personal journal, blog post, novel, short story, etc.)	1	12	4.75	3.25	10.59	64
6	Playing a musical instrument	1	14	4.9	3.22	10.39	40
7	Painting or drawing	1	14	5.16	2.75	7.58	83
8	Gardening or attending to indoor plants	1	14	5.17	3.3	10.9	42
9	Reading novels, stories, poetry or play	1	14	5.22	2.88	8.28	72
10	Rehearsing or performing in a play, drama, opera, musical theatre	1	17	5.25	4.62	21.35	12
11	Learning or practising magic tricks or circus skills	2	11	5.33	4.03	16.22	3
12	Watching films or TV shows	1	13	5.42	2.91	8.45	108
13	Composing music or songs (including electronic/computer music)	1	13	5.77	3.16	9.99	22
14	Creating artworks or animations on a computer	1	14	5.96	3.69	13.64	25
15	Textile crafts such as embroidery, crocheting, knitting	1	13	5.97	2.99	8.93	29
16	Making films or videos	1	15	6.39	3.42	11.69	33
17	Playing video games	1	16	6.39	3.27	10.69	57
18	Fashion or costume design	2	13	6.5	3.16	10	8
19	Jewellery making	4	9	6.57	1.68	2.82	7
20	Sculpture	2	11	6.6	3.01	9.04	5
21	Calligraphy	1	11	6.88	3.41	11.61	8
22	Wood crafts such as carving or furniture making	3	10	7	3.08	9.5	4
23	Viewing and contemplating artworks (e.g., in books or online)	2	15	7.28	2.58	6.67	43
24	Pottery or ceramics	1	12	7.33	3.45	11.89	6
25	Photography	2	16	7.43	3.52	12.36	35
26	Make-up artistry	3	15	7.52	3.44	11.81	23
**Participants aged 25–34 (*n* = 127)**							
1	Listening to music	1	11	3.23	2.03	4.14	100
2	Playing a musical instrument	1	9	3.88	2.55	6.48	32
3	Dancing	1	12	3.91	2.54	6.43	23
4	Singing	1	13	3.97	2.86	8.16	31
5	Wood crafts such as carving or furniture making	1	9	4	2.86	8.2	10
6	Gardening or attending to indoor plants	1	14	4.12	3.02	9.14	58
7	Composing music or songs (including electronic/computer music)	1	8	4.17	2.27	5.14	6
8	Pottery or ceramics	2	10	4.63	2.39	5.73	8
9	Fashion or costume design	1	16	4.64	4.01	16.05	11
10	Painting or drawing	1	12	4.66	2.55	6.52	61
11	Making films or videos	1	9	4.71	2.15	4.63	14
12	Textile crafts such as embroidery, crocheting, knitting	1	12	4.79	2.9	8.43	38
13	Cookery or baking	1	15	4.79	2.97	8.84	84
14	Reading novels, stories, poetry or play	1	12	4.93	2.57	6.58	73
15	Calligraphy	1	11	5	3.27	10.67	6
16	Creative writing (e.g., personal journal, blog post, novel, short story, etc.)	1	13	5.12	3	9.01	42
17	Playing video games	1	12	5.12	3.19	10.2	43
18	Watching films or TV shows	1	13	5.2	2.88	8.28	102
19	Creating artworks or animations on a computer	1	13	5.27	3.52	12.38	11
20	Jewellery making	1	13	5.7	3.93	15.41	10
21	Sculpture	2	11	6.13	3.02	9.11	8
22	Learning or practising magic tricks or circus skills	1	11	6.25	4.15	17.19	8
23	Photography	1	14	6.36	3.44	11.84	36
24	Make-up artistry	2	15	7.67	4.08	16.67	9
25	Rehearsing or performing in a play, drama, opera, musical theatre	6	10	8	1.63	2.67	3
26	Viewing and contemplating artworks (e.g., in books or online)	5	14	8.26	2.33	5.45	35
**Participants aged 35–54 (*n* = 294)**							
1	Listening to music	1	13	3.49	2.14	4.56	200
2	Painting or drawing	1	14	3.62	2.9	8.39	130
3	Singing	1	15	4.04	2.83	8.01	82
4	Gardening or attending to indoor plants	1	12	4.04	2.28	5.18	169
5	Dancing	1	14	4.16	2.79	7.78	62
6	Textile crafts such as embroidery, crocheting, knitting	1	13	4.24	3.07	9.4	115
7	Playing a musical instrument	1	14	4.46	3.07	9.4	59
8	Pottery or ceramics	1	11	4.47	3.57	12.78	19
9	Composing music or songs (including electronic/computer music)	1	16	4.68	3.3	10.86	28
10	Creative writing (e.g., personal journal, blog post, novel, short story, etc.)	1	15	4.92	2.96	8.79	101
11	Cookery or baking	1	15	5.1	3.09	9.52	184
12	Sculpture	1	12	5.19	3.33	11.09	32
13	Rehearsing or performing in a play, drama, opera, musical theatre	2	17	5.36	4.05	16.41	11
14	Reading novels, stories, poetry or play	1	18	5.36	3.16	10.01	184
15	Fashion or costume design	2	11	5.71	2.73	7.46	24
16	Calligraphy	4	12	5.86	2.7	7.27	7
17	Learning or practising magic tricks or circus skills	1	14	5.9	4.48	20.09	10
18	Watching films or TV shows	1	15	5.99	2.95	8.71	226
19	Photography	1	15	6.11	3.47	12.04	96
20	Playing video games	1	17	6.25	3.81	14.49	59
21	Making films or videos	1	16	6.61	3.15	9.92	44
22	Wood crafts such as carving or furniture making	3	14	6.71	2.86	8.2	14
23	Creating artworks or animations on a computer	2	14	6.83	2.7	7.31	41
24	Viewing and contemplating artworks (e.g., in books or online)	2	16	7.13	2.87	8.25	119
25	Jewellery making	1	13	7.76	3.26	10.65	17
26	Make-up artistry	3	16	8.22	4.24	17.95	9
**Participants aged 55+ (*n* = 105)**							
1	Fashion or costume design	1	7	3.57	2.19	4.82	7
2	Singing	1	12	3.63	2.78	7.73	16
3	Textile crafts such as embroidery, crocheting, knitting	1	15	3.63	3.04	9.26	41
4	Gardening or attending to indoor plants	1	14	3.86	3.06	9.36	63
5	Painting or drawing	1	11	3.87	2.76	7.59	46
6	Listening to music	1	11	3.94	2.45	6.02	54
7	Wood crafts such as carving or furniture making	2	7	4.17	1.67	2.81	6
8	Playing a musical instrument	1	10	4.33	3.14	9.89	12
9	Creative writing (e.g., personal journal, blog post, novel, short story, etc.)	1	14	4.5	2.62	6.88	32
10	Dancing	1	8	4.6	2.73	7.44	10
11	Reading novels, stories, poetry or play	1	13	4.84	2.67	7.1	62
12	Cookery or baking	1	13	4.98	2.99	8.93	59
13	Composing music or songs (including electronic/computer music)	1	9	5.25	3.34	11.19	4
14	Watching films or TV shows	1	16	5.55	3	9.01	73
15	Sculpture	1	11	6	3.26	10.6	10
16	Photography	1	13	6.22	3.17	10.05	32
17	Creating artworks or animations on a computer	1	14	6.31	3.89	15.14	13
18	Viewing and contemplating artworks (e.g., in books or online)	2	13	6.45	2.76	7.6	40
19	Pottery or ceramics	1	14	6.57	3.96	15.67	7
20	Make-up artistry	7	7	7	0	0	1
21	Making films or videos	1	15	7.2	4.66	21.76	5
22	Playing video games	1	15	7.71	4.33	18.78	7
23	Jewellery making	2	11	7.82	2.55	6.51	11
24	Calligraphy	7	12	9.5	2.5	6.25	2
25	Learning or practising magic tricks or circus skills	13	13	13	0	0	1
26	Rehearsing or performing in a play, drama, opera, musical theatre	0	0	0	0	0	0

“Min. Rank” refers to the lowest ranking that this ACA received, whereas “Max. Rank” refers to the highest ranking that this ACA received. ACAs are ranked by the Mean of the reported rank of effectiveness for each category.

In comparison to the above three age groups, the 55+ group did not list “Listening to music” as their most effective ACA for making them “feel better,” which was instead ranked 6th. The 55+ group listed “Fashion or costume design” as their most effective ACA, which by comparison was ranked between 18th and 9th place in the younger groups. “Singing” nevertheless still ranked as the second most effective ACA, but as this was the only music-related ACA ranked in the top five, this reflects a substantial difference in ACA engagement by age group. Similarly, “Dancing” was ranked substantially lower at 10th place.

**Figure 1:**
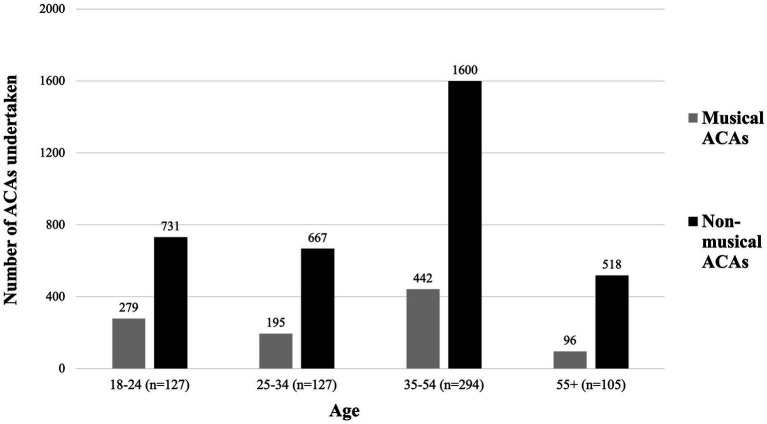
Count of undertaken ACAs across the four age groups. ACAs are split into (1) Music related ACAs, and (2) All other ACAs (i.e., those not related to music).

To summarize these results, we broadly grouped all ACAs into two groups: musical and non-musical. The musical ACAs consisted of “Listening to music,” “Singing,” “Playing a musical instrument”; “Composing music or songs”; “Dancing”; “Rehearsing or performing in a play, drama, opera, musical” (N.B. the authors acknowledge that “Dancing” and “Rehearsing or performing in a play, drama, opera, musical” do not always occur with music, but, considering the frequency with which they do, it was deemed most appropriate to classify these as musical rather than non-musical). This summary of ACAs by music relation, across the four age groups, is shown in Figure 1. While the 18–24 age group (*n* = 127) reported undertaking 1,010 ACAs in total, 279 (27.6%) of these were musical. Similarly, the 25–34 age group (*n* = 127) reported undertaking 862 ACAs in total, with 195 (22.6%) of these being musical and the 35–54 age group (*n* = 294) reported undertaking 2042 ACAs, 442 of these (21.6%) were musical. In contrast, the 55+ age group (*n* = 105) reported undertaking 614 ACAs, of which 96 (15.6%) were musical. This constitutes a substantial decrease, not only in the ranking of musical ACAs as the age of participants increased, but also in their general prevalence. Next, we examine responses to the MusEQ subscales, to further understand this difference and the possible reasons for it.

### Examination of MusEQ subscales by age group

**Figure 2:**
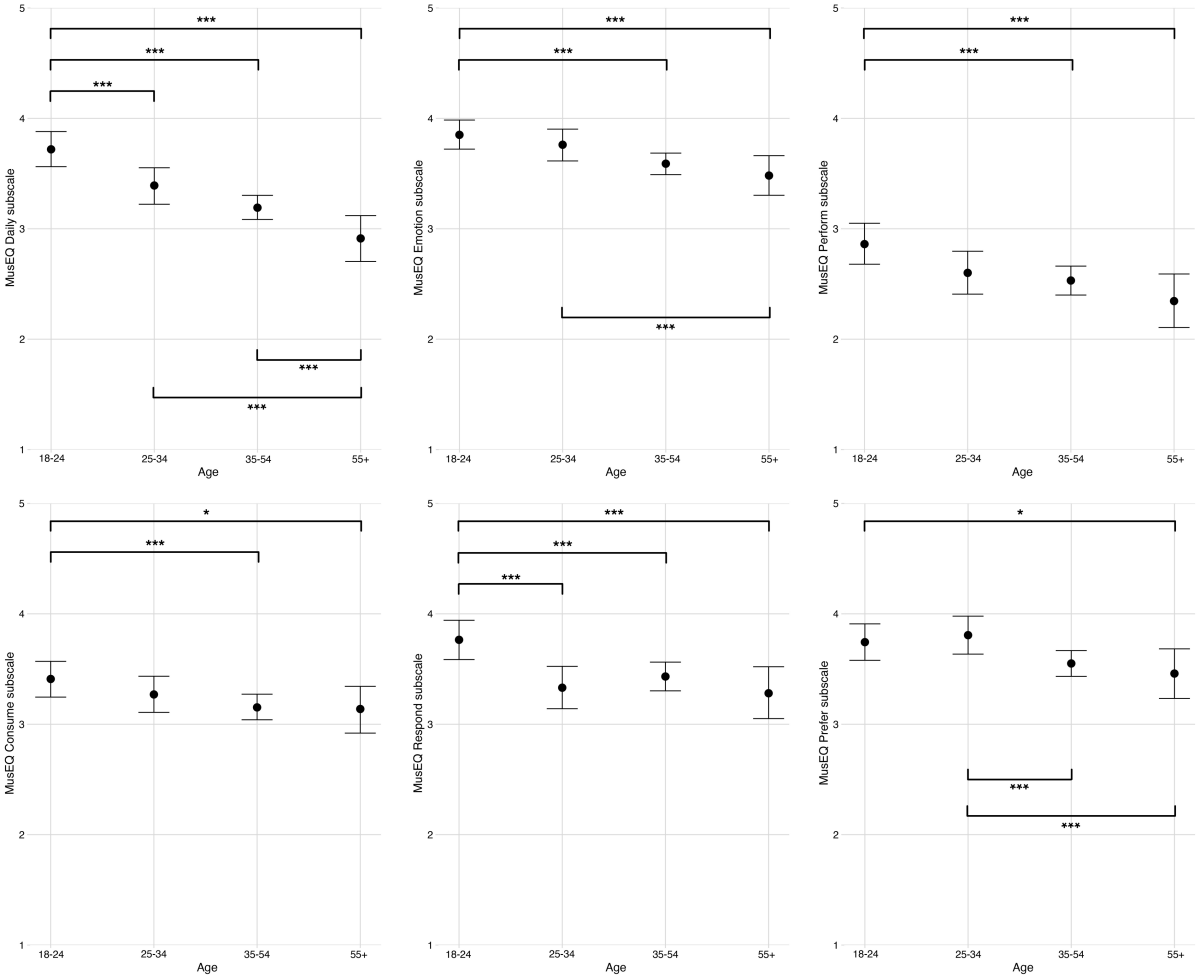
Ratings of the six MusEQ subscales, split across the four age groups. Error bars are 95% Bayesian credibility intervals. Additionally, evidence ratios 39–60 are denoted with *, evidence ratios 61–90 are denoted with **, and evidence ratios >90 are denoted with ***.

The six MusEQ subscales were only completed by participants who reported undertaking at least one musical ACA (as defined above). This led to MusEQ responses by 508 (77.8%) of participants. Analysis of these data was performed with Bayesian modelling, produced in *R* (version 4.1.2) using the *brms* package (Bürkner, 2017). Each Bayesian model contained a separate MusEQ subscale as the dependent variable, and the four age groups as the independent variable. Bayesian modelling was chosen as it is robust against uneven group sizes (as is the case between age groups). Additionally, instead of simply testing a null hypothesis (as is done *via* inferential statistics, with a *p* value) Bayesian modelling provides a strength of evidence for the presence or absence of an effect. Therefore, instead of producing a *p* value Bayesian models produce an evidence ratio, in which a result close to 0 constitutes the least amount of evidence for an effect/difference, and a higher evidence ratio corresponds to greater evidence for an effect/difference. We provide additional information and references to interpreting Bayesian evidence ratios within the Supplementary Material, but for the analyses concerning MusEQ an evidence ratio greater than or equal to 39 can be considered strong evidence for a difference in ratings, and equivalent to a *p* value <0.05 (Makowski et al., 2019). Findings for the six Bayesian models are plotted in Figure 2, and we have indicated the equivalent of significance where appropriate. Additionally, within the Supplementary Material we report a) exact *M* and *SD* values for each MusEQ subscale separated by age group within Supplementary Table 1, and b) exact evidence ratios for each comparison within Supplementary Table 2.

Generally speaking, the ratings in Figure 2 outline a trend of decreasing musical engagement as the age of participants increased. For all six subscales there was strong evidence (equivalent of *p* < 0.05) that the youngest age group produced higher ratings (i.e., stronger musical engagement) than the older adults group (55+). This trend was particularly pronounced for the subscales “Daily,” “Emotion,” “Perform” and “Respond.” For the “Daily” subscale, there was very strong evidence (evidence ratio > 90) that there was a difference between the youngest age group (18–24) and every other age group. At the same time, for this scale there was also evidence of the same strength of a difference between the older adults group and every other age group, with the younger participant groups scoring much more highly than the older participants on this subscale. This finding suggests that music plays a role in the daily lives of the younger participants significantly more so than it does for the older participants. For the “Emotion” subscale, a similar trend was observed in that there was very strong evidence (evidence ratio > 90) that the youngest age group (18–24) scored much more highly than the two oldest age groups (35–54, 55+), while the oldest participants (55+) scored significantly lower (evidence ratio > 90) than the 25–34 age group. This suggests that younger participants also used music to regulate emotion much more so than older participants. For the “Perform” subscale, there was again very strong evidence (evidence ratio > 90) that the youngest participants scored more highly than the two oldest age groups (35–54, 55+), suggesting that younger participants were much more likely to engage in musical performance and to use music to negotiate their sense of identity than older participants. Similarly, for the “Respond” subscale, the youngest participants (18–24) scored significantly higher (evidence ratio > 90) than every other age group, suggesting that participants in this age group are much more likely to respond to music in an embodied way (e.g., singing along, humming, foot taping) than older participants. Based on these findings, we can conclude that younger participants (and particularly the younger adults group, aged 18–24) reported significantly stronger musical engagement than older participants along all six subscales, with the strongest differences being along the “Daily,” “Emotion,” “Perform” and “Respond” subscales.

### Examination of mental health by demographic and protective variables

**Figure 3:**
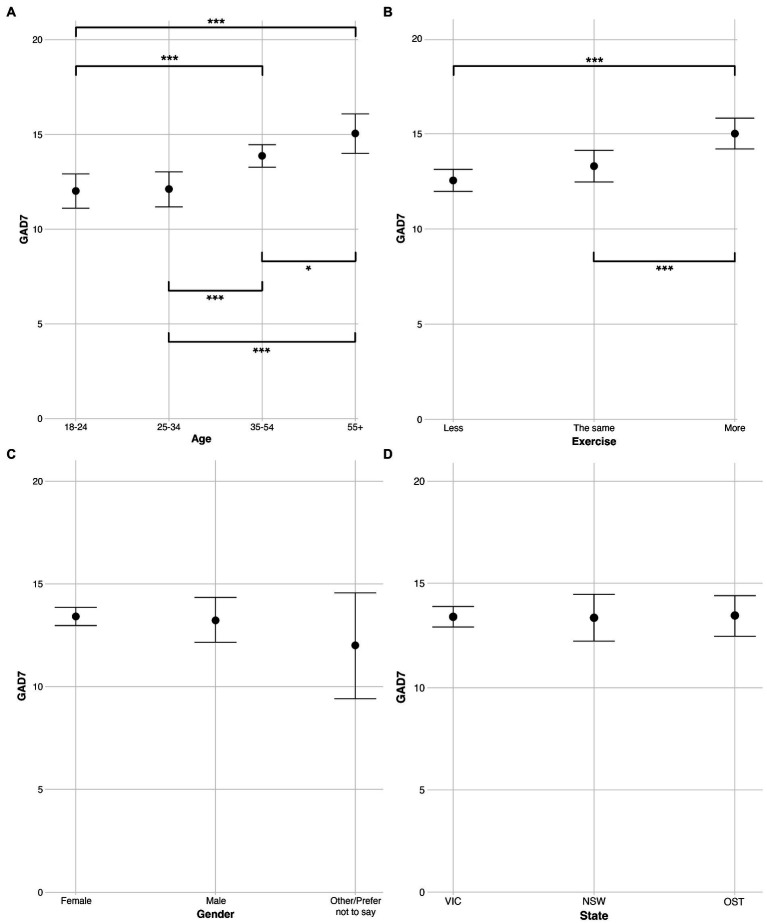
Ratings of GAD7 (anxiety) split across levels of the independent variables **(A)** age, **(B)** exercise, **(C)** gender, and **(D)** state. Error bars are 95% Bayesian credibility intervals. Additionally, evidence ratios 39–60 are denoted with *, evidence ratios 61–90 are denoted with **, and evidence ratios >90 are denoted with ***.

**Figure 4:**
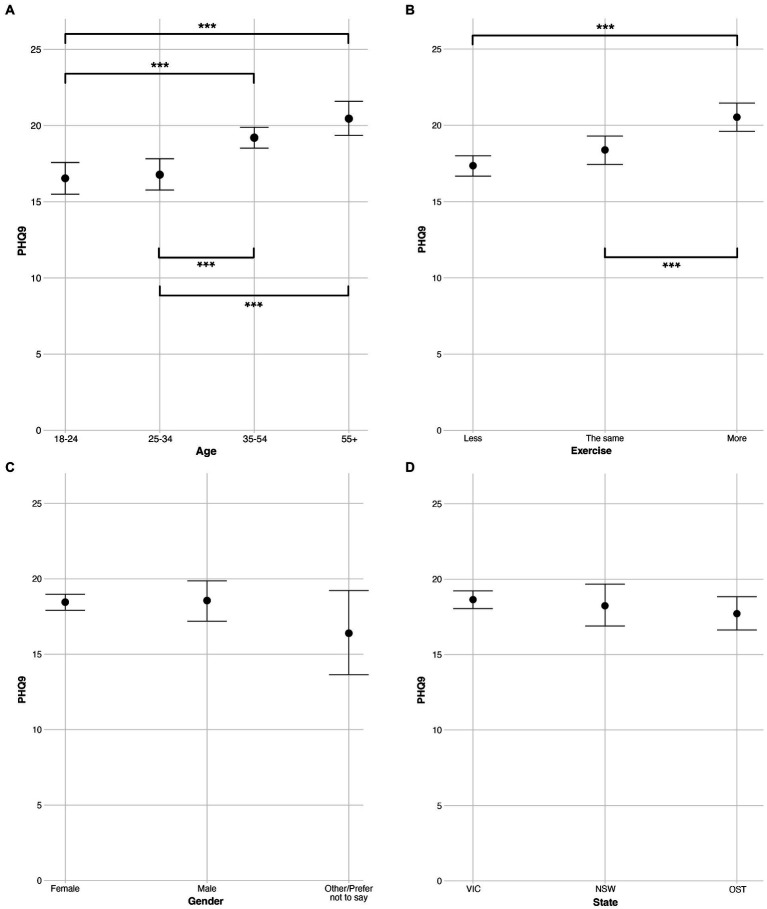
Ratings of PHQ9 (depression) split across levels of the independent variables **(A)** age, **(B)** exercise, **(C)** gender, and **(D)** state. Error bars are 95% Bayesian credibility intervals. Additionally, evidence ratios 39–60 are denoted with *, evidence ratios 61–90 are denoted with **, and evidence ratios >90 are denoted with ***.

Next, we examine ratings of anxiety (GAD7) and depression (PHQ9) for each participant, and specifically, how differences by age group, exercise, gender, and state predict these mental health ratings. Cronbach’s alpha between GAD7 and PHQ9 ratings was calculated as 86, indicating a high level of consistency between the two mental health variables. As above, these data were analyzed with Bayesian modelling to counteract differences in sample size (most notably in age and gender groups). Eight separate models were run, with four concerning anxiety and four concerning depression. Each model contained a separate independent variable (age, exercise, gender, or state) and was subjected to the earlier evidence ratio threshold of 39. Ratings for the four GAD7 models are plotted in Figure 3, and ratings for the four PHQ9 models are plotted in Figure 4. As above, within the Supplementary Material we report a) exact *M* and *SD* values for each model, separated by the independent variables within Supplementary Table 3, and b) exact evidence ratios for each comparison within Supplementary Table 4 (GAD7) and Supplementary Table 5 (PHQ9).

#### Examination by age

Regarding age, both models (Figures 3A, 4A) produced a trend in which older participants reported higher ratings (i.e., reported feeling less anxious and depressed) than younger participants. When examining specific comparisons, for both models there was strong evidence (evidence ratio > 90) of the two oldest age groups faring better than both the youngest age group (18–24) and the younger established adults group (25–34). There were no differences in anxiety or depression between the 18–24 and 25–34 groups, indicating that, among our participants, these primary differences in mental health during the pandemic occurred above the age of 35 (i.e., in the more established adults). In summary, the models depict age as a robust predictor of anxiety and depression with those in the youngest two groups, below the age of 35, reporting experiencing significantly worse anxiety and depression during the pandemic.

#### Examination by exercise

Regarding exercise, both models (Figures 3B, 4B) produced strong evidence (evidence ratio > 90) that those exercising more fared better than those exercising in either of the other two groups (those exercising less, and those exercising the same). Thus, these models suggest that increased exercise is a robust and effective predictor of anxiety and depression among our participants.

#### Examinations by gender and state

For the models regarding gender (Figures 3C, 4C) and state (Figures 3D, 4D) no comparisons produced an evidence ratio above 12. In sum, there is little evidence to suggest a meaningful difference in anxiety or depression by either gender or state.

## Discussion

This study investigated the ways young adults in Australia (aged 18–24) used artistic creative activities (ACAs) to support their mental health during the pandemic lockdowns of 2020, as well as potential differences in the use of music and the arts for this purpose across different age groups (18–24, 25–34, 35–54, 55+). It also explored whether specific variables were strong predictors of the mental health of participants. We asked three specific research questions:RQ1: asked about the ACAs that made participants in each age group “feel better” during the pandemic;RQ2: asked whether similarities or differences could be observed in the ways participants in each age group used music during the pandemic;RQ3: asked whether any or all of four diverse variables (age, exercise, gender, and state) predicted variances in mental health symptoms (specifically anxiety and depression).Based on the findings of previous research, we also made three hypotheses:

*H1*: that music listening would rank as the most effective ACA for making participants “feel better” during the pandemic in each age group.

*H2*: that the youngest participants (aged 18–24) would be more likely to use music for the purposes of identity expression than older participants.

*H3*: that young adults, women, and residents of the state Victoria would report poorer mental health (anxiety and depression) than other participants.

With regard to RQ1, the findings showed that music listening was ranked as the most effective activity for making participants “feel better” for the three youngest age groups (18–24, 25–34, 35–54), but not for the older adults group (55+). As such, H1 was only partially supported. This drop in the effectiveness of music listening as an emotion regulation strategy for the older adult participants was further supported by a broader observed trend in which musical activities, with the exception of singing, were reported to be less effective as the age of participants increased, with the oldest participants favoring non-musical activities such as fashion and costume design, textile crafts, gardening, and painting over musical activities. Similarly, dancing featured highly among the effectiveness rankings in all age groups except the oldest (55+), which supports emerging research that—much like increased exercise—dancing can lead to both physical and psychological benefits (Conceição et al., 2016; Muro and Artero, 2017; Staiano et al., 2017). The fact that the oldest age group did not rate dancing among their most effective ACAs for making them “feel better” may be due to the fact that dancing requires greater physical exertion that may not appeal to older adults (physical activity declines with age, but significant decline typically occurs from age 55; see Fan et al., 2013). These findings highlight that forms of engagement in music and the arts for the purposes of emotion regulation during the pandemic differed by age group, and also that singing was universally viewed as effective, with each age group ranking singing in fourth place or higher. This lends support to previous research that has shown singing, and especially group singing, to be an important strategy for maintaining mental health and wellbeing into older age (Lee et al., 2016). This also suggests that increasing pandemic-safe opportunities for singing during future crises may be effective in supporting mental health regardless of age, while other musical activities may also offer additional support, particularly for people aged under 55.

This point was further elaborated by the analyses of responses to the MusEQ measure (Vanstone et al., 2016), which, with regard to RQ2, highlighted the trend of decreasing musical engagement as the age of participants increased, while also strongly emphasizing the importance of music engagement for the young adults group (18–24). These young adult participants reported significantly stronger musical engagement scores than older groups on all six of the MusEQ subscales. This was especially the case for the “Daily,” “Emotion,” “Perform” and “Respond” subscales, where the evidence of difference between the youngest age group and older age groups was the strongest. This finding supported H2, but also provided substantial new insights into the role of music in the lives of young adults aged 18–24 during the pandemic beyond the negotiation and expression of identity (Tarrant et al., 2002; O’Neill, 2017; Levstek and Banerjee, 2021). These findings showed that young adult participants aged 18–24:

1. integrated music into the routine aspects of their daily lives much more so than participants in all other age groups (for example, listening to music while doing chores, cooking, or when feeling bored);
2. used music to regulate emotion much more so than participants aged 35 or older, for example, for the purposes of relaxing, cheering themselves up, or recalling memories from the past;
3. engaged in performance-based musical activities such as playing a musical instrument for pleasure or otherwise creating their own music, and also used music to negotiate their sense of identity, much more so than participants aged 35 or older;
4. used music to negotiate and express a sense of social identity, for example, by using music to facilitate social interaction or to identify themselves as a “musical” person, much more so than participants aged 35 or older;
5. were much more likely to respond to music in embodied ways, for example, by singing along to heard music, or humming or foot tapping, than participants in every other age group.

These findings build on previous literature which underlines the variety of roles and functions music can play in everyday life (DeNora, 2000; Chin et al., 2018; Krause et al., 2018), but extends this by revealing how people of different ages have used music differently in the context of a pandemic lockdown. It suggests that music has been a particularly important resource (an “aesthetic technology”; see Krueger, 2019) for young Australians during the COVID-19 pandemic, which they have put to work in specific ways in order to manage their mental health. Given that young adults were among the most severely impacted by the pandemic in terms of mental health (e.g., see Biddle et al., 2022), these insights should provide a useful basis for determining how specific types of musical activity can better support young adults in future crises or contexts in which they are experiencing social isolation (which was the case during the COVID-19 pandemic in Australia; see Smith and Lim, 2020). Specifically, this might include finding new ways to:

1. support young people in their attempts to fold musical activities into the routine flow of daily life, and exploring new ways that this might be done;
2. ensure that young people have opportunities to experiment with different ways of experiencing music, and to consider their emotional implications (e.g., whether this makes them feel more cheerful, relaxed, connected to others, and the like);
3. encourage a variety of active music-making activities alongside passive music listening as an important creative outlet for young people, and to experiment with different forms of music making beyond formal/institutional music training;
4. support musical activities that allow young people to safely connect with others, and through this, to explore their own identities in relation to others (this includes musical identities such as “musician” as well as other social identities that may emerge through identification with specific social groups);
5. support activities that allow musical experiences to be embodied in creative ways that go beyond the passive experience of music listening, for example, by integrating music with other forms of creative expression such as movement, dance, design, visual art/craft, or visual digital media.

Indeed, age is not the only variable that has been shown to interact with MusEQ ratings. Kiernan et al. (2021) reported significant negative relationships between anxiety (measured with GAD7) and the MusEQ subscales, as well as between depression (measured with PHQ9) and the MusEQ subscales. These negative relationships indicated that as participants reported experiencing increasing symptoms of anxiety and depression, they also tended to report engaging more with music (although any causality or direction of this relationship is not necessarily implied). This notion of music being a companion through stressful and lonely times is not novel (Garrido and Schubert, 2011), although these prior findings indicate that there may be a number of variables interacting with engagement in music (as measured through MusEQ, here). Therefore, future work should aim to examine a wider range of interactions with the MusEQ subscales, including but not limited to age and mental health.

Regarding RQ3, the findings showed that age and exercise were robust predictors of mental health in our participants, with participants aged 34 or under, and participants exercising less or the same amount than they had been prior to the onset of the pandemic, reporting the worst symptoms of anxiety and depression. However, it is important to note we are not able to infer causation between exercise and mental health; it is possible that some or all of our participants who reported faring better were simply motivated to do more exercise, and subsequent research should aim to properly explore the direction of this relationship. Regardless, these findings strengthen the existing literature that suggests increased exercise to be a particularly robust and effective method for offsetting detriments to mental health and wellbeing during a crisis (e.g., Colley et al., 2020; Coyle et al., 2021; Faulkner et al., 2021). Indeed, this finding regarding exercise may be particularly useful for helping younger people offset some of the additional psychosocial impacts they may have been experiencing during this time. Our analyses also revealed little evidence that symptoms of anxiety and depression differed by either gender or state. With this in mind, H3 was only supported regarding the variable age, whereas it was rejected regarding the variables gender and state.

Given that the existing research literature has strongly emphasized that women are among those whose mental health was impacted the worst during the pandemic (Biddle et al., 2022), our finding concerning this is surprising. However, some discrepancies in the literature do exist. Shi et al. (2020) collected anxiety and depression ratings in China from March to May 2020, and found that men displayed substantially higher risk of depression than women, and no differences in anxiety by gender. Additionally, studies on anxiety and depression in China from February 2020 (Fu et al., 2020), in Italy from April to May 2020 (Gualano et al., 2020), and in the UK in March 2020 (Shevlin et al., 2020) each observed significantly higher levels of anxiety in women, yet no observed differences in depression by gender. Building on this literature, one interpretation of our findings is that further investigation may be needed into the ways that local, culturally specific gender roles (as opposed to biological sex) may have played a role in impacting mental health during this pandemic crisis. Moreover, additional focus is needed on non-binary categories, as studies suggest people who do not identify comfortably with binary gender categories may also be at risk (Restar et al., 2021). The present study was not able to examine in detail the mental health of people who identify as non-binary, due to the small number of participants in our study who identified in this way. Furthermore, given that Victoria endured by far the longest and strictest lockdowns of any state or territory in Australia (Knowlton, 2022), it was also surprising that no variations were found in ratings of anxiety and depression by state. However, this may be explained, at least in part, by the fact that Australia was not impacted by widespread COVID-19 infections until comparatively late in 2020 (Berger and Reupert, 2020; Pew Research Center, 2020). In other words, this could be a by-product of the fact that the present survey commenced relatively early into the first Australian lockdown period of 2020, whereas it is possible that subsequent surveys would have shown clearer differences in mental health ratings by gender and state.

While this work was able to highlight novel aspects relating to Australian mental health during COVID-19, and specifically how this related to engagement with ACAs by age group, it was not without its limitations. First, due to the terms of our Human Research Ethics Approval we were unable to examine responses for participants under the age of 18. This meant that, much like many of the studies cited in our Introductory section, our youngest examined age group ranged only by 6 years (18–24), whereas the other age groups ranged by 10 or more years (e.g., 25–34). The decision was made to retain this smaller age group of 18–24, based on the prevalence of similar approaches in the literature, although this could alternatively be addressed with the inclusion of participants under 18 years, or with the use of smaller age brackets. Indeed, the inclusion of younger participants would provide additional insight and may well lead to additional observed differences, and we encourage future studies to explore this aspect. Second, the recruitment period for this study spanned almost 5 months. This approach was taken to maximize the number of participants collected and to provide an overview of these understudied research question, although it may also have had an impact on the findings as various participants completed the survey during different times of the lockdown period. Future work should aim to control for this to a greater degree.

Third, our recruitment strategy of widely disseminating an online public survey was not able to attract even numbers of participants for all independent variable groups. This was most pronounced for participants who were above 65 years of age, those in the “Other/Prefer not to say” gender group, and those within the “OST” state group (“Other States and Territories”). As noted in the Materials and Methods section, we grouped the two oldest age groups (creating a 55+ age group) to counteract this smaller sample of participants above 65 years, and selected an analysis method (Bayesian modelling) that is robust against mismatched variable categories. Regardless, no strong evidence was observed for examinations of gender and state. The lack of observed differences between groups of gender and state may be explained, at least in part, by these smaller sample sizes, and with this in mind future work should aim to recruit a more diverse and balanced sample, if possible. It must also be noted that the variable exercise only contained three levels (exercising less, the same, or more) and so provides limited insight. A more comprehensive approach—such as one estimating exercise hours over the course of the pandemic—may be able to provide a more detailed understanding of the relationship between exercise and mental health during this time. This was beyond the scope of this study, which simply aimed to provide an initial overview of observed relationships.

Additionally, due to the multifaceted and subjective nature of engagement with ACAs, there are some aspects of this engagement that a general survey will undoubtedly be unable to cover in detail. The inclusion of other methodological approaches and paradigms—such as qualitative ethnographic research and practice-based research—are essential to the research landscape of arts and health and should be encouraged in the future (Clift et al., 2021; Grebosz-Haring et al., 2022). We also note that our comparison of the prevalence of ACAs by various age groups was based solely on the Mean of the Rank score, which does not constitute an inferential analysis. We were not able to find an appropriate inferential test in which the assumptions matched our collected data, referring to the number of categories (26 ACAs) and the number of variable levels (i.e., four between-subjects groups of age). While we suggest that the method used here is reasonably robust and fitting for this purpose, future studies may be able to design their surveys in a manner that better allows the implementation of inferential testing (such as a Chi-squared test) than was possible here. Finally, while our approach was aimed to give a general snapshot of Australian mental health, and how this related to age and ACAs during the 2020 lockdowns, we acknowledge that an alternative approach—such as one containing a longitudinal design—may well yield different, and potentially more important findings. We recommend such an approach for studies of this kind in future crises.

## Conclusion

We conclude that significant differences have been observed in the ways people of different age groups have engaged with music and the arts during the COVID-19 pandemic in Australia to support their mental health. Young adults (aged 18–24) overwhelmingly favored musical activities while older adult participants (aged 55+) favored non-musical activities. Furthermore, we have identified specific ways in which music supported the mental health of young adults during the pandemic, and suggest that these may provide a useful foundation for strategic future policy and public health decisions regarding the involvement of music and the arts for mental health promotion during future crises. We also conclude that, amongst our participants, age and exercise were robust predictors of mental health, while gender and state/territory of residence were not, and this finding suggests interesting avenues for further research.

## Data availability statement

The raw data supporting the conclusions of this article will be made available by the authors, without undue reservation.

## Ethics statement

The studies involving human participants were reviewed and approved by Human Research Ethics Committee of the University of Melbourne. The patients/participants provided their written informed consent to participate in this study.

## Author contributions

All authors contributed to the creation of this work, including the design of the project. Manuscript write-up and analyses were led by AC, with support from the other authors. AC and FK led data gathering. All authors contributed to the article and approved the submitted version.

## Funding

This work was funded by the Creativity and Wellbeing Hallmark Research Initiative at the University of Melbourne (Grant holder JD).

## Conflict of interest

The authors declare that this research was conducted in the absence of any commercial or financial relationships that could be construed as a potential conflict of interest.

## Publisher’s note

All claims expressed in this article are solely those of the authors and do not necessarily represent those of their affiliated organizations, or those of the publisher, the editors and the reviewers. Any product that may be evaluated in this article, or claim that may be made by its manufacturer, is not guaranteed or endorsed by the publisher.

## Supplementary material

The Supplementary material for this article can be found online at: https://www.frontiersin.org/articles/10.3389/fpsyg.2022.993259/full#supplementary-material

## Footnotes

^1^In this work we use the term "young adults" to refer to those aged approximately 18 to 24. This definition is based upon previous work such as by Biddle et al. (2022), and Czeisler et al. (2020). Other authors such as Dale et al. (2021) use similar terms such as "younger people" to describe those in the same age bracket. This can be seen in contrast to "established adults," which we use to refer to all people approximately 25 years and above, based on similar empirical comparisons by Gilleen et al. (2021), Kassim et al. (2022), and Zolopa et al. (2022), among others.

^2^The authors acknowledge that there is some debate on what constitutes an ACA, and that not all readers may agree with our included 26 ACAs. This list of ACAs was formulated based on the most frequently cited literature (e.g., Fancourt et al., 2019) and the most recent literature (e.g., Vidas et al., 2021; Drake et al., 2022), as well as activities that had been frequently posted about in online media at the time of lockdown, in an effort to capture the most accurate comparison available.
