# Supplementary material for: Creativity in lockdown: Understanding how music and the arts supported mental health during the COVID-19 pandemic by age group
Source: Front Psychol. 2022 Oct 6;13:993259. doi: 10.3389/fpsyg.2022.993259 (PMC9583145; doi:10.3389/fpsyg.2022.993259)
Supplement: Supplementary file 1 [file Data_Sheet_1.pdf]

Supplementary Material

Creativity in lockdown: Understanding how music and the arts can support mental health by age group

Frontiers in Psychology, 2022

Anthony Chmiel, Frederic Kiernan, Sandra Garrido, Sarah Lensen, Martha Hickey, & Jane W. Davidson

doi: 10.3389/fpsyg.2022.993259

Overview and breakdown of ethnicity, income levels, and infection levels in NSW and VIC

This document was compiled to facilitate understanding of the primary document, while stopping the primary document from becoming overly lengthy. We begin this Supplementary Material document by providing the breakdowns of ethnicity, income levels, and COVID-19 infection levels in the states New South Wales (NSW) and Victoria (VIC), as well as for Australia overall. In June 2020, Australia contained a population of approximately 25.7 million, with the two most populous states being NSW and VIC[1]. Overall in Australia at this time, the five largest ethnic groups were English (approximately 33%); Australian (approximately 30%), Irish (approximately 9.5%), Scottish (approximately 8.5%), and Chinese (approximately 5.5%)[2].

In 2020 NSW contained 8.1 million residents, with the five most largest ethnic groups being Australian (approximately 30%), English (approximately 30%), Irish (approximately 10%), Scottish (approximately 8%), and Chinese (approximately 7%)[3]. The most commonly spoken languages at home, apart from English, were Mandarin (approximately 3%), Arabic (approximately 3%), Cantonese (approximately 2%), Vietnamese (approximately 1.5%), and Hindi (approximately 1%), whereas close to 70% of households only used English at home[3]. Additionally, the Mean weekly

---

[1] *Australian Bureau of Statistics*. (2020). https://www.abs.gov.au/statistics/people/population/national-state-and-territory-population/jun-2020

[2] *Australian Bureau of Statistics*. (2020). https://www.abs.gov.au/census/find-census-data/quickstats/2021/AUS

[3] *Australian Bureau of Statistics*. (2021). https://www.abs.gov.au/census/find-census-data/quickstats/2021/1

income for NSW for an adult working full-time was $1,751.90 AUD[4]. This is in comparison to the national Mean weekly income at $1,711.60 AUD[4]. By the end of May, 2020, NSW had just over 3,000 confirmed infection cases of COVID-19, and this had increased to more than 4,300 cases by the end of October 2020[5]. This was in comparison with approximately 7,200 COVID-19 cases in the entire country at the start of June 2020, and almost 28,000 cases by the end of October 2020.

In 2020, VIC contained 8.1 million residents, with approximately 28% of these listed as "Australian" heritage, approximately 30% of these listed as "English" heritage, approximately 10% as "Irish" heritage, approximately 8% as "Scottish", and approximately 6% as "Chinese"[3]. The most commonly spoken languages at home, apart from English, were Mandarin (approximately 3%), Vietnamese (approximately 2%), Greek (approximately 2%), Italian (approximately 2%), and Punjabi (approximately 1%), whereas close to 70% of households only used English at home[3]. Additionally, the Median weekly household income for NSW for an adult working full-time was $1,718.50 AUD[4]. By the start of June, 2020, VIC had 1,645 confirmed infection cases of COVID-19, although this had increased to more than 20,000 cases by the end of October 2020[6].

Information relating to Bayesian modelling and evidence ratios

In this section we discuss Bayesian modelling, as is used in the primary document. All Bayesian models were analyzed in *R* using the *brms* package[7,8] which is a front end for the Bayesian inference and Markov Chain Monte Carlo (MCMC) sampler *Stan*[9].

Bayesian modelling was chosen for many of the analyses in this work as it can have distinct advantages over inferential statistics (e.g., ANOVAs and the like). Most notably, this approach can be more robust against differences in sample sizes between variable levels (as is particularly the case in the present study between age groups and gender groups).

To interpret Bayesian modelling, the strength of evidence for each comparison is provided by an evidence ratio, which is the posterior odds of the effect being in the direction specified in the tested

---

[4] *Australian Bureau of Statistics*. (2020). https://www.abs.gov.au/statistics/labour/earnings-and-working-conditions/average-weekly-earnings-australia/nov-2020

[5] *NSW Health*. (2020). https://www.health.nsw.gov.au/news/Pages/20201116_02.aspx

[6] *Victoria State Government, Health and Human Services*. (2020). https://www.dhhs.vic.gov.au/coronavirus-update-victoria-30-october-2020

[7] Bürkner, P.-C. (2017). brms: An R package for Bayesian multilevel models using Stan. *Journal of Statistical Software*, *80*(1), 1-28.

[8] Bürkner, P.-C. (2018). Advanced Bayesian multilevel modeling with the R package brms. *The R Journal*, *10*(1), 395-411.

[9] Carpenter, B., Gelman, A., Hoffman, M. D., Lee, D., Goodrich, B., Betancourt, M., Brubaker, M., Guo, J., Li, P., & Riddell, A. (2017). Stan: A probabilistic programming language. *Journal of Statistical Software*, *76*(1), 1-32.

comparison. As detailed in the primary document, we consider an evidence ratio greater than or equal to 39 to be strong, and loosely analogous to a two-tailed *p*-value below .05[10]. Therefore, in the following tables that contain evidence ratios, we have indicated an asterisk next to all evidence ratios ≥39 to indicate an 'equivalence of inferential statistical significance'. Similarly, in Figures 2, 3, and 4 within the primary document we have denoted comparisons with a strong evidence ratio with significance equivalents. That is, we have denoted comparisons with an evidence ratio 39-60 with *, evidence ratios with 60-90 with **, and evidence ratios >90 with ***. In this work we do not consider any evidence ratios below 39 as substantial support for a meaningful difference, although we will note that at times in the broader literature other authors will sometimes consider evidence ratios approaching 39 (e.g., those above 30) as "medium support" (much like a "marginally significant finding", i.e., *p* values approaching .05.). Based on this information, readers without prior knowledge of Bayesian modelling should be able to achieve a required understanding of the Bayesian hypothesis tests within the primary document, and to make general comparisons between the reported evidence ratios and what their inferential (*p* value) equivalents would be.

Supplementary Table 1

Descriptive statistics (M and SD) for the six MusEQ subscales, split by the four age groups. The "Overall" row refers to all age groups collapsed. Ratings ranged from 1 to 5.

MusEQ Subscale	Age group	Overall	18-24	25-34	35-54	55+
Daily	*M*	3.3	3.7	3.4	3.2	2.9
	SD	0.9	0.9	0.9	0.9	0.8
Emotion	*M*	3.7	3.9	3.8	3.6	3.5
	SD	0.7	0.7	0.7	0.8	0.7
Perform	*M*	2.6	2.9	2.6	2.5	2.3
	SD	1.0	1.1	1.0	1.0	1.0
Consume	*M*	3.2	3.4	3.3	3.1	3.1
	SD	0.9	0.8	0.9	0.9	0.8
Respond	*M*	3.5	3.8	3.3	3.4	3.2
	SD	1.0	0.9	1.0	1.0	1.0
Prefer	*M*	3.6	3.7	3.8	3.5	3.5
	SD	0.9	0.9	0.8	0.9	1.0

Note. Sample size is 508, as only participants who reported undertaking at least one music-related ACA completed the MusEQ scale. Music-related ACAs were "Listening to music", "Singing", "Playing a musical instrument"; "Composing music or songs"; "Dancing"; and "Rehearsing or performing in a play, drama, opera, musical".

[10] Makowski, D., Ben-Shachar, M. S., Chen, S. H. A., & Lüdecke, D. (2019). Indices of existence and significance in the Bayesian framework. *Frontiers in Psychology*, *10*, 2767.

Supplementary Table 2

*Output for the six Bayesian models concerning the MusEQ subscales, split by the four age groups. The arrow between groups indicates the direction of the tested hypothesis. Comparisons with an evidence ratio above 39 (indicating strong evidence) are denoted with *.*

Model	Compared groups	Evidence ratio	Posterior probability	C.I. lower	C.I. upper	
MusEQ Daily subscale						
	18-24 > 25-34	1332.33	1.00	-0.52	-0.14	*
	18-24 > 35-54	Infinite	1.00	-0.69	-0.36	*
	18-24 > 55+	Infinite	1.00	-1.03	-0.59	*
	25-34 > 35-54	34.71	0.97	-0.37	-0.03	
	25-34 > 55+	1332.33	1.00	-0.70	-0.26	*
	35-44 > 55+	110.11	0.99	-0.48	-0.09	*
MusEQ Emotion subscale						
	18-24 > 25-34	4.42	0.82	-0.26	0.07	
	18-24 > 35-54	570.43	1.00	-0.40	-0.12	*
	18-24 > 55+	1332.33	1.00	-0.56	-0.17	*
	25-34 > 35-54	37.10	0.97	-0.32	-0.02	
	25-34 > 55+	96.56	0.99	-0.48	-0.08	*
	35-44 > 55+	5.25	0.84	-0.28	0.06	
MusEQ Perform subscale						
	18-24 > 25-34	35.04	0.97	-0.48	-0.04	
	18-24 > 35-54	799.00	1.00	-0.52	-0.14	*
	18-24 > 55+	1332.33	1.00	-0.77	-0.26	*
	25-34 > 35-54	2.58	0.72	-0.27	0.13	
	25-34 > 55+	16.94	0.94	-0.51	0.01	
	35-44 > 55+	9.72	0.91	-0.41	0.05	
MusEQ Consume subscale						
	18-24 > 25-34	7.55	0.88	-0.33	0.05	
	18-24 > 35-54	172.91	0.99	-0.42	-0.09	*
	18-24 > 55+	45.51	0.98	-0.50	-0.05	*
	25-34 > 35-54	6.80	0.87	-0.28	0.05	
	25-34 > 55+	5.11	0.84	-0.36	0.08	
	35-44 > 55+	1.24	0.55	-0.22	0.18	

MusEQ Respond subscale						
	18-24 > 25-34	399.00	1.00	-0.65	-0.22	*
	18-24 > 35-54	499.00	1.00	-0.51	-0.15	*
	18-24 > 55+	1999.00	1.00	-0.73	-0.23	*
	25-34 > 35-54	4.00	0.80	-0.09	0.29	
	25-34 > 55+	1.66	0.62	-0.30	0.20	
	35-44 > 55+	5.92	0.86	-0.38	0.07	
MusEQ Prefer subscale						
	18-24 > 25-34	2.40	0.71	-0.13	0.26	
	18-24 > 35-54	33.48	0.97	-0.36	-0.03	
	18-24 > 55+	44.98	0.98	-0.51	-0.06	*
	25-34 > 35-54	124.00	0.99	-0.44	-0.08	*
	25-34 > 55+	152.85	0.99	-0.58	-0.11	*
	35-44 > 55+	3.21	0.76	-0.30	0.13	

Note. Evidence ratio = Posterior probability / (1 – Posterior probability). C. I. Lower and C. I. Upper give 90% Bayesian credibility intervals. An Evidence ratio threshold of 39 is used to infer the equivalent of a statistically significant difference at $p < .05$.

Supplementary Table 3

Descriptive statistics (M and SD) for the GAD7 and PHQ9 scales, split by groups within the independent variables (age, exercise, gender, and state). GAD7 ranged from 0 to 21, and PHQ9 ranged from 0 to 27. In each case, 0 reflects feeling the worst (i.e., the most anxiety or depression).

**Independent variable**	**Independent variable groups**	*n*	**GAD7 (anxiety)**		**PHQ9 (depression)**	
			M	*SD*	*M*	*SD*
Age	18-24	127	11.9	5.1	16.3	6.2
	25-34	127	12.1	5.6	16.8	6.8
	35-54	294	13.9	5.4	19.2	6.0
	55+	105	15.1	5.3	20.6	5.7
Exercise	18-34	327	12.5	5.7	17.3	6.6
	18-34	161	13.3	5.3	18.4	6.3
	18-34	165	15.1	4.6	20.6	5.3
Gender	Female	551	13.4	5.3	18.5	6.3
	Male	87	13.2	6.2	18.5	6.6
	Other/Pref.	15	11.7	6.8	15.8	6.5
State	VIC	446	13.3	5.6	18.6	6.3
	NSW	86	13.3	5.2	18.2	6.5
	OST	121	13.4	5.3	17.7	6.4

Note. The “Other/Prefer not to say” gender group has been abbreviated to “Other/Pref.”

Supplementary Table 4

*Output for the four GAD7 Bayesian models. The arrow between groups indicates the direction of the tested hypothesis. Comparisons with an evidence ratio 39 and above (indicating strong evidence) are denoted with *.*

Model	Compared groups	Evidence ratio	Posterior probability	C.I. lower	C.I. upper	
GAD7, age						
	25-34 > 18-24	1.22	0.55	-0.97	1.17	
	35-54 > 18-24	Infinite	1.00	0.95	2.75	*
	55+ > 18-24	Infinite	1.00	1.92	4.18	*
	35-54 > 25-34	1332.33	1.00	0.86	2.66	*
	55+ > 25-34	Infinite	1.00	1.78	4.10	*
	55+ > 35-54	41.11	0.98	0.21	2.17	*
GAD7, exercise						
	The same > Less	14.04	0.93	-0.08	1.58	
	More > Less	Infinite	1.00	1.62	3.28	*
	More > The same	443.44	1.00	0.73	2.65	*
GAD7, gender						
	Male > Female	0.57	0.36	-1.20	0.78	
	Female > Other/Pref.	6.41	0.86	-3.61	0.71	
	Male > Other/Pref.	4.29	0.81	-1.18	3.58	
GAD7, state						
	NSW > VIC	0.87	0.47	-1.03	0.96	
	OST > VIC	1.31	0.57	-0.79	0.97	
	OST > NSW	1.31	0.57	-1.13	1.36	

Note. Evidence ratio = Posterior probability / (1 – Posterior probability). C. I. Lower and C. I. Upper give 90% Bayesian credibility intervals. An Evidence ratio threshold of 39 is used to infer the equivalent of a statistically significant difference at $p < .05$. The “Other/Prefer not to say” gender group has been abbreviated to “Other/Pref.”

Supplementary Table 5

*Output for the four PHQ9 Bayesian models. The arrow between groups indicates the direction of the tested hypothesis. Comparisons with an evidence ratio 39 and above (indicating strong evidence) are denoted with *.*

Model	Compared groups	Evidence ratio	Posterior probability	C.I. lower	C.I. upper	
PHQ9, age						
	25-34 > 18-24	1.69	0.63	-0.93	1.45	
	35-54 > 18-24	Infinite	1.00	1.66	3.69	*
	55+ > 18-24	Infinite	1.00	2.64	5.17	*
	35-54 > 25-34	3999.00	1.00	0.86	2.66	*
	55+ > 25-34	Infinite	1.00	2.35	4.94	*
	55+ > 35-54	31.52	0.98	0.15	2.33	
PHQ9, exercise						
	The same > Less	21.10	0.95	0.02	1.98	
	More > Less	Infinite	1.00	2.23	4.07	*
	More > The same	1332.33	1.00	1.04	3.24	*
PHQ9, gender						
	Male > Female	1.21	0.55	-1.03	1.26	
	Female > Other/Pref.	11.74	0.92	-4.39	0.31	
	Male > Other/Pref.	10.30	0.91	-0.46	4.75	
PHQ9, state						
	NSW > VIC	0.37	0.27	-1.57	0.76	
	OST > VIC	0.07	0.06	-1.95	0.09	
	OST > NSW	0.38	0.28	-1.88	0.93	

Note. Evidence ratio = Posterior probability / (1 – Posterior probability). C. I. Lower and C. I. Upper give 90% Bayesian credibility intervals. An Evidence ratio threshold of 39 is used to infer the equivalent of a statistically significant difference at $p < .05$. The “Other/Prefer not to say” gender group has been abbreviated to “Other/Pref.”
